# Stand-Off Detection of Alcohol Vapors Exhaled by Humans

**DOI:** 10.3390/s18051310

**Published:** 2018-04-24

**Authors:** Jaroslaw Mlynczak, Jan Kubicki, Krzysztof Kopczynski

**Affiliations:** Institute of Optoelectronics, Military University of Technology, Gen. Witolda Urbanowicza 2, 00-908 Warsaw, Poland; jan.kubicki@wat.edu.pl (J.K.); krzysztof.kopczynski@wat.edu.pl (K.K.)

**Keywords:** alcohol detection, stand-off detection, alcohol detection in humans

## Abstract

Early detection of humans under the influence of alcohol in public places (workplace, public gathering) is particularly important for safety reasons. In this article, the theoretical analysis of stand-off detection of alcohol in the air exhaled by humans as well as experimental results of the developed experimental setup is presented. The concept of differential absorption of two laser beams at different wavelengths was used. The idea of using standard deviation of the relative difference of the amplitudes of two signals to detect the alcohol was applied for the first time. The idea was verified by the experiments and it was shown that a reliable device can be developed that can efficiently detect alcohol concentration in the exhaled air at the level of 0.3 mg/L (0.63‰). Moreover, the concept of such device examining humans entering a specific area was proposed. The results of this article may be useful to scientists or engineers working on alcohol detection in human blood.

## 1. Introduction

Stand-off detection of vapors and gases utilizing their resonance absorption of electromagnetic radiation is well-known and widely described in literature [1,2,3,4,5,6,7]. A particular case of this method is the detection of alcohol in the air exhaled by humans. This issue is especially important in a situation where a person, being under the influence of alcohol, may cause a serious or even fatal accident in a workplace or during mass gatherings such as football matches or concerts. Currently used techniques for screening humans for the presence of alcohol in their blood may be divided into two types. One of them is a special device that analyses the air blown into them, called breathalyzers, that are widely used by the police. The second one involves the testing of blood samples. Although the blood test is socially regarded as more reliable, both techniques are equally effective, which can be proved by Henry’s Law saying that in a specific temperature the alcohol vapor pressure above the alcohol solution corresponds to the alcohol concentration in this solution [8]. Thus, there are many different breathalyzers that measure the concentration of alcohol in the exhaled air and determine the insobriety of humans [9]. However, the use of these devices is time consuming and discomforts people who are examined. The application of stand-off detection of alcohol in the air exhaled by humans may solve the problem. There are some articles describing stand-off detection of alcohol in moving cars [10,11,12,13], however its application to examine humans entering a specific area has not been described so far. In this article the theoretical analysis of such application as well as experimental results of the developed experimental setup are presented. The idea of using standard deviation of the relative difference of the amplitudes of two signals to detect the alcohol was applied for the first time. Moreover, the concept of the device for stand-off detection of alcohol in the air exhaled by humans entering a specific area was proposed. The proposed system would not be comparable in cost to existing systems, and is not looking to replace them, but rather, is designed while keeping in mind a very specific application.

## 2. Materials and Methods

The method proposed for stand-off detection of alcohol in the air exhaled by humans is based on the absorption of a monochromatic electromagnetic radiation (laser beam) tuned to the absorption band of the alcohol.

Intensity of the radiation beam that goes through an absorbing medium I(v) (with no saturation) can be described by Beer–Lamber law in the following form:

(1) $$I\left(v\right)=I_{0}\left(v\right)\cdot e^{-\alpha \left(v\right)\cdot c\cdot l}$$

where:

$I_{0}\left(v\right)$ (W)—intensity of the beam entering the medium.
α(v) (dm^3^∙g^−1^∙cm^−1^)—volume-mass absorption coefficient of the medium.
c (g∙dm^−3^)—mass concentration of the medium.
l (cm)—thickness of the medium.
v (Hz)—frequency of radiation.

If the absorbing medium is the alcohol exhaled by a person its mass concentration is spatially inhomogeneous and it changes with time t, thus c=c(x,y,z,t), where x, y and z are space coordinates.

Usually the laser beam cross section is much smaller than the absorbing medium. Thus, the mass concentration of the medium can be considered as constant in x, y plane perpendicular to the propagation direction of the beam z and c(x, y, z, t) can be replaced by c(z, t). The average mass concentration of the medium $\hat{c}\left(t\right)$ can be expressed as:

(2) $$\hat{c}\left(t\right)=\frac{1}{l}\int_{0}^{l}c\left(z,t\right)dz$$

Thus, the Equation (1) takes the form:

(3) $$I\left(v,t\right)=I_{0}\left(v\right)\cdot e^{-\alpha \left(v\right)\cdot \hat{c}\left(t\right)\cdot l}$$

To eliminate the impact of the environment on the intensity of the laser beam absorbed by the medium, a second laser beam with wavelength close to but outside of the absorption band of the absorbing medium should be applied. The intensities of both laser beams that go through the absorbing medium $I_{1}\left(t\right)$ (monitoring beam) and $I_{2}\left(t\right)$ (reference beam) can be expressed as:

(4) $$I_{1}\left(t\right)=I_{01}\cdot e^{-\alpha_{1}\cdot \hat{c}\left(t\right)\cdot l}$$

(5) $$I_{2}\left(t\right)=I_{02}\cdot e^{-\alpha_{2}\cdot \hat{c}\left(t\right)\cdot l}$$

where:

$I_{01}$ (W)—intensity of the monitoring beam entering the medium.
$I_{02}$ (W)—intensity of the reference beam entering the medium.
$\alpha_{1}$ (dm^3^∙g^−1^∙cm^−1^)—volume-mass absorption coefficient of the medium for the monitoring beam.
$\alpha_{2}$ (dm^3^∙g^−1^∙cm^−1^)—volume-mass absorption coefficient of the medium for the reference beam.

To effectively compare the transmission of the laser beams by the absorbing medium, the intensities of both beams entering the medium should be the same. Such situation can be achieved by normalization of $I_{1}\left(t\right)$ to $I_{2}\left(t\right)$. Thus, the expression for normalized $I_{1}\left(t\right)$ can be written as:

(6) $$I_{1n}\left(t\right)=k\cdot I_{01}\cdot e^{-\alpha_{1}\cdot \hat{c}\left(t\right)\cdot l}$$

where:

(7) $$k=\frac{I_{02}}{I_{01}}$$

Working with a linear detector, the intensities $I_{1n}\left(t\right)$ and $I_{2}\left(t\right)$ can be replaced by generated signals $S_{1}\left(t\right)$ and $S_{2}\left(t\right)$. Thus normalized $S_{1}\left(t\right)$ and $S_{2}\left(t\right)$ can be described by the following equations:

(8) $$S_{1n}\left(t\right)=k\cdot S_{1}\left(t\right)=k\cdot S_{01}\cdot e^{-\alpha_{1}\cdot \hat{c}\left(t\right)\cdot l}$$

(9) $$S_{2}\left(t\right)=S_{02}\cdot e^{-\alpha_{2}\cdot \hat{c}\left(t\right)\cdot l}$$

where:

(10) $$k=\frac{S_{02}}{S_{01}}$$

The coefficient k can be determined by measuring $S_{1}\left(t\right)$ and $S_{2}\left(t\right)$ without the absorbing medium. Then, $S_{1}\left(t\right)=S_{01}$ and $S_{2}\left(t\right)=S_{02}$, hence $k=\frac{S_{2}\left(t\right)}{S_{1}\left(t\right)}$.

To effectively detect the alcohol in exhaled air the relative difference of the signals $S_{2}\left(t\right)$ and $S_{1n}\left(t\right)$ can be utilized and for laser sources characterized by high long-term power stability it can be expressed as:

(11) $$\Delta s\left(t\right)=\frac{S_{2}\left(t\right)-S_{1n}\left(t\right)}{S_{2}\left(t\right)}=\frac{S_{02}\cdot e^{-\alpha_{2}\cdot \hat{c}\left(t\right)\cdot l}-S_{01}\cdot \frac{S_{02}}{S_{01}}\cdot e^{-\alpha_{1}\cdot \hat{c}\left(t\right)\cdot l}}{S_{02}\cdot e^{-\alpha_{2}\cdot \hat{c}\left(t\right)\cdot l}}=\frac{e^{-\alpha_{2}\cdot \hat{c}\left(t\right)\cdot l}-e^{-\alpha_{1}\cdot \hat{c}\left(t\right)\cdot l}}{e^{-\alpha_{2}\cdot \hat{c}\left(t\right)\cdot l}}=1-e^{-\left(\alpha_{1}-\alpha_{2}\right)\cdot \hat{c}\left(t\right)\cdot l}$$

In case if there is no alcohol $\hat{c}\left(t\right)=0$ and Δs(t)=0, however if there is alcohol $\alpha_{2}<\alpha_{1}$ and 0<Δs(t)<1. For a given concentration of alcohol the value of Δs(t) can be increased by increasing $\alpha_{1}-\alpha_{2}$ applying lasers operating at appropriate wavelengths or by increasing l applying multiple transition of the laser beams through the absorbing medium.

The problem is that in real environment the concentration $\hat{c}\left(t\right)$ changes very much in time which is caused by the exhalation and gusts of air. The changes are stochastic and, because of exponential relation, have strong impact on Δs(t). A stable situation is only possible when $\hat{c}\left(t\right)=0$. However, the fluctuations of $\hat{c}\left(t\right)$ can be utilized to detect the alcohol in exhaled air. From Equation (11) the value of Δs(t) depends on $\hat{c}\left(t\right)$, thus the higher fluctuation of $\hat{c}\left(t\right)$ the higher fluctuations of Δs(t). In turn, fluctuations of $\hat{c}\left(t\right)$ depend on alcohol concentration in the exhaled air and are higher for higher alcohol concentration. The value of fluctuation of Δs(t) for subsequent measurements can be expressed by its standard deviation σ. Thus, for higher alcohol concentration one can expect higher σ.

Lasers available on the market, that can be used for the described application, are usually characterized by limited long-term power stability. It leads to the situation when the coefficient k, determined at the beginning of the measurements for a situation without the absorbing medium, becomes invalid with the passing of time. Thus, the relative difference of the signals Δs(t), calculated using Equation (11) is incorrect. The problem can be solved by replacing the coefficient k by $k_{r}$ according to the following equation:

(12) $$k_{r}=\frac{\hat{S_{2}}\left(t\right)}{\hat{S_{1}}\left(t\right)}$$

$\hat{S_{1}}\left(t\right)$ and $\hat{S_{2}}\left(t\right)$ are signals $S_{1}\left(t\right)$ and $S_{2}\left(t\right)$ measured and averaged over a period in which the changes of the laser power are small in comparison to the changes of alcohol concentration (alcohol concentration changes should be faster than laser power changes).

Therefore, the normalized signal from the detector $S_{1}\left(t\right)$ can be written as:

(13) $$S_{1r}\left(t\right)=k_{r}\cdot S_{1}\left(t\right)$$

Therefore, the relative difference of the signals $S_{2}\left(t\right)$ and $S_{1p}\left(t\right)$, for subsequent measurements in the defined period, can be expressed as:

(14) $$\begin{array}{l} \Delta s_{r}\left(t\right)=\frac{S_{2}\left(t\right)-S_{1r}\left(t\right)}{S_{2}\left(t\right)}=\frac{S_{02}\cdot e^{-\alpha_{2}\cdot \hat{c}\left(t\right)\cdot l}-S_{01}\cdot \frac{\hat{S_{2}}\left(t\right)}{\hat{S_{1}}\left(t\right)}\cdot e^{-\alpha_{1}\cdot \hat{c}\left(t\right)\cdot l}}{S_{02}\cdot e^{-\alpha_{2}\cdot \hat{c}\left(t\right)\cdot l}}\\ \;=\frac{e^{-\alpha_{2}\cdot \hat{c}\left(t\right)\cdot l}-\frac{S_{01}}{S_{02}}\cdot \frac{\hat{S_{2}}\left(t\right)}{\hat{S_{1}}\left(t\right)}\cdot e^{-\alpha_{1}\cdot \hat{c}\left(t\right)\cdot l}}{e^{-\alpha_{2}\cdot \hat{c}\left(t\right)\cdot l}}\\ \;=1-\frac{S_{01}}{S_{02}}\cdot \frac{\hat{S_{2}}\left(t\right)}{\hat{S_{1}}\left(t\right)}\cdot e^{-(\alpha_{1}-\alpha_{2})\cdot \hat{c}\left(t\right)\cdot l} \end{array}$$

Because the value of $k_{r}$ are determined for signals $S_{1}\left(t\right)$ and $S_{2}\left(t\right)$ that can be measured with the presence of absorbing medium the value of $\Delta s_{r}\left(t\right)$ can be positive as well as negative for different measurements (oscillates around zero). However, the changes of the concentration $\hat{c}\left(t\right)$ still have the impact on it as in case of Equation (11). It means that the standard deviation $\sigma_{r}$ of the distribution of $\Delta s_{r}\left(t\right)$, for subsequent measurements in the defined period, can be utilized to detect the alcohol in the exhaled air.

## 3. Experiment and Results

**Figure 1:**
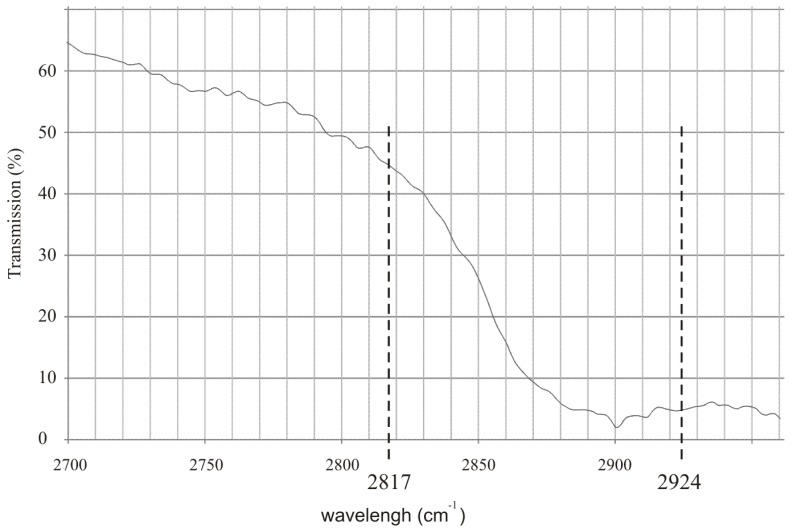
Experimental transmission spectrum of the rectified spirit along with the wavelengths of commercially available inter-band cascade lasers.

To effectively detect alcohol in the exhaled air, lasers that operate at appropriate wavelengths must be selected. Commercially available inter-band cascade lasers operating at 3.42 µm (2924 cm^−1^) and 3.55 µm (2817 cm^−1^) seem to be an excellent choice. In Figure 1 the experimental transmission spectrum of the rectified spirit along with the wavelengths of the mentioned lasers are presented. It is shown that the difference between absorption coefficient of the alcohol at both wavelengths is relatively high which is desirable for effective detection.

**Figure 2:**
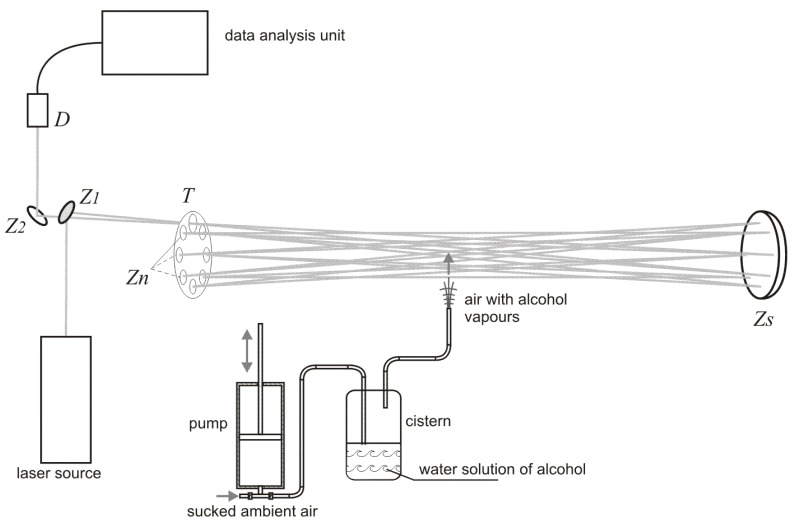
Experimental setup used for investigation of alcohol detection in the air exhaled by humans.

The experimental setup used for investigation of alcohol detection in the air exhaled by humans is presented in Figure 2. The radiation beam from the laser source was directed by the plane mirror Z1 (diameter 12.7 mm) to the multiple reflection system consisting of seven small plane mirrors Zn (diameter 12.7 mm) and one big concave mirror Zs (diameter 40 mm, focal length 6 m). The mirrors Zn were mounted on the disc T and could be independently adjusted. After 16 transitions through the multiple reflection system the beam was reflected by the plane mirror Z2 (diameter 12.7 mm) and was absorbed by the detector D. The signals from the detector were transmitted to the data analysis unit consisting of a custom-built electronic board and a computer. The air containing alcohol was blown into the multiple reflection system by the simulator of a drunken person that consisted of an air pump and a cistern containing a water solution of alcohol. The pump pumped the air of volume 0.5 l with a frequency of 20 times per minute through the cistern. The air going out from the cistern contained alcohol vapors with concentration that depended on the concentration of alcohol in water solution. The exact value of the alcohol vapors concentration was measured by a commercially available professional breathalyzer A2.0/04 produced by AWAT company (Warsaw, Poland).

**Figure 3:**
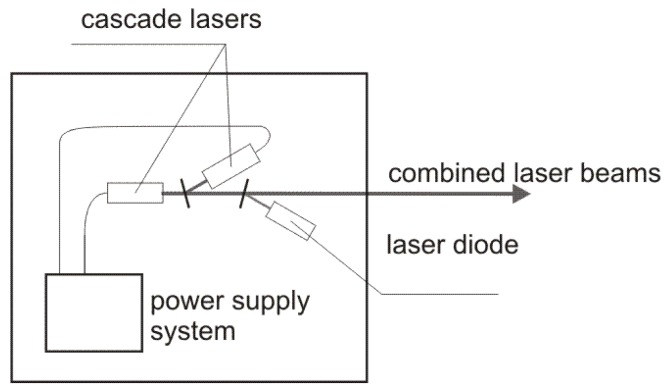
Diagram of the laser source.

The diagram of the laser source is presented in Figure 3. Two inter-band cascade lasers IF3420CM1 i IF3550CM1 operating at 3.42 µm and 3.55 µm wavelength with output power of ~20 mW developed by Thorlabs (Newton, NJ, USA) were used. The lasers were modulated with the frequency of 50 and 80 kHz, respectively. The duty cycle of the modulation was 50%. The beams of the cascade lasers were combined into one single beam by a plane-parallel plate. To enable an effective alignment of the experimental setup a laser diode operating at red wavelength was also integrated into the laser source by second plane-parallel plate.

A custom-built detector HgCdTe developed by VIGO System (Warsaw, Poland) was used. The signal from the detector was transmitted to the custom-built electronic board developed by INTSOL (Warsaw, Poland) company where it was sampled with the frequency of 1.25 GHz. Every 5 ms the signal with the same period of 5 ms was analyzed by a computer (FFT analysis) and the amplitudes at the lasers modulation frequency were calculated. The values of these amplitudes were registered by the computer in the period of 3.5 s giving 700 different values of the amplitudes for each laser.

The main experiment was preceded by investigation of the laser power stability of the cascade lasers. Every minute 700 values of the amplitudes of the signal at the lasers modulation frequency were calculated and averaged. The results are presented in Table 1 where $\hat{S_{1}}$ and $\hat{S_{2}}$ are averaged amplitudes of the signal for lasers operating at 3.42 µm and 3.55 µm, respectively. The values are expressed in relative units (r.u.) using only 8 significant digits. These values are proportional to the signals from the detector.

Relatively slight changes of the laser powers may seem to be negligible for most applications, however for the detection of slight changes of the alcohol vapor transmission they may be indeed important.

In the experiment four water solution with different concentration of alcohol were investigated. The exact concentration of alcohol was measured by the breathalyzer. For each alcohol concentration three independent measurements, when the alcohol vapors were blown into the multiple reflection system, were done. To determine the k coefficient, before each measurement, additional three measurements when the alcohol vapours were not blown into the multiple reflection system were carried out. The results are presented in Table 2, where numbers 01, 02 and 03 represent the results without the alcohol vapors while numbers 1, 2 and 3 represent the results with the alcohol vapors. The symbols in the table represent:

*c*—concentration of alcohol vapors in the air blown into the multiple reflection system.
$\hat{S_{1}}$—amplitude averaged over 700 values for laser operating at 3.42 µm wavelength.
$\hat{S_{2}}$—amplitude averaged over 700 values for laser operating at 3.55 µm wavelength.
$k=\frac{\hat{S_{2}}}{\hat{S_{1}}}$—calculated for the situation when the alcohol vapors were not blown into the multiple reflection system.
$\hat{\Delta s}$—relative difference of the amplitudes calculated according to Equation (11) and averaged over 700 values.
$\bar{\Delta s}$—value of $\hat{\Delta s}$ averaged over three measurements.
σ—standard deviation of $\bar{\Delta s}$.
$\frac{\sigma }{\bar{\Delta s}}$—relative standard deviation of $\Delta \hat{s}$ for three measurements.

**Figure 4:**
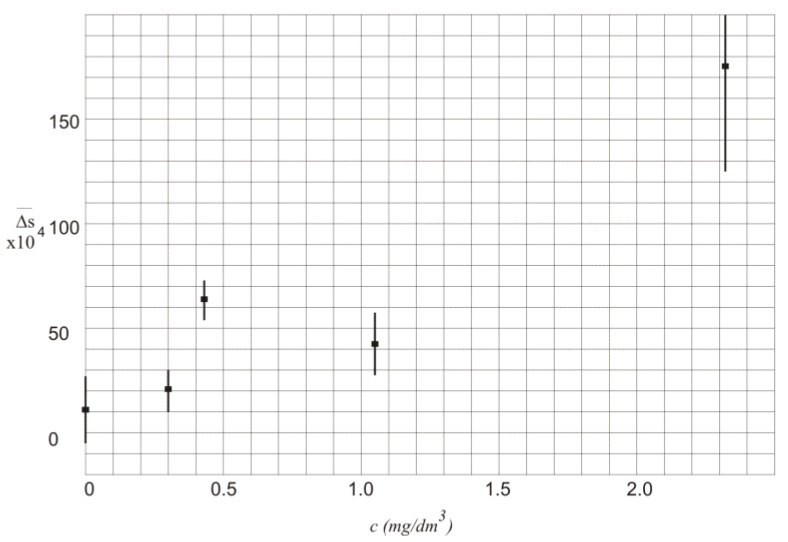
Values of $\bar{\Delta s}$ in function of alcohol concentration *c*.

The values of $\bar{\Delta s}$ in function of alcohol concentration *c* along with the standard deviation σ are also presented in Figure 4.

In Table 2 and Figure 4 the values of $\bar{\Delta s}$ are characterized by high standard deviation that mainly results from instability of the laser powers. Thus, the measurement of the exact value of the alcohol concentration is not possible.

Using the same 700 values of amplitudes of signals $S_{1}\left(t\right)$ and $S_{2}\left(t\right)$, as in Table 2, the values of relative difference of the signals $\Delta s_{r}\left(t\right)$ were calculated according to the Equation (14). For calculated values of $\Delta s_{r}\left(t\right)$ standard deviation $\sigma_{r}$ was estimated. The results of calculations are presented in Table 3, where:

$\hat{\Delta s_{r}}$—relative difference of the amplitudes calculated according to Equation (14) and averaged over 700 values.
$k_{r}=\frac{\hat{S_{2}}\left(t\right)}{\hat{S_{1}}\left(t\right)}$—for the situation when the alcohol vapours were blown into the multiple reflection system.
$\sigma_{r}$—standard deviation of $\hat{\Delta s_{r}}$.
$\bar{\sigma_{r}}$—value of $\sigma_{r}$ averaged over three measurements.
$\Delta \bar{\sigma_{r}}$—standard deviation of $\bar{\sigma_{r}}$.
$\frac{\Delta \bar{\sigma_{r}}}{\bar{\sigma_{r}}}$—relative standard deviation of $\bar{\sigma_{r}}$.

**Figure 5:**
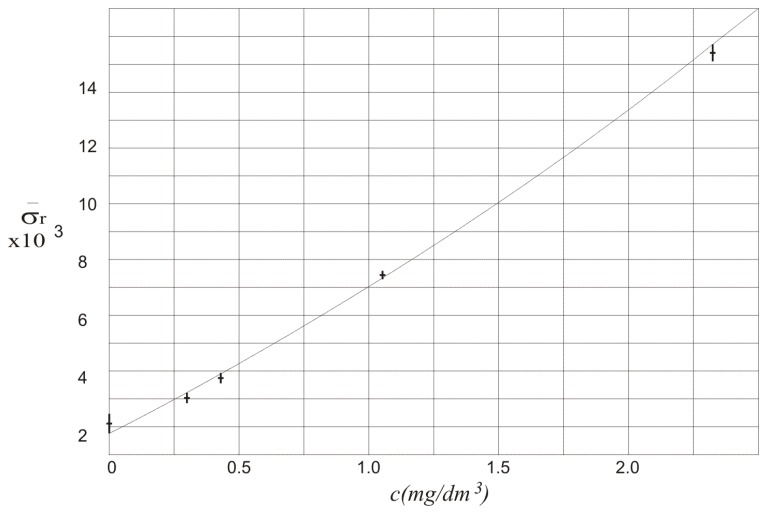
Values of $\bar{\sigma_{r}}$ in function of alcohol concentration *c*.

The results of calculation of $\bar{\sigma_{r}}$ in function of alcohol concentration *c* are also presented in Figure 5. The relation between $\bar{\sigma_{r}}$ and alcohol concentration is almost linear. The standard deviation of the relative difference of the amplitudes of the measured signals $S_{1}\left(t\right)$ and $S_{2}\left(t\right)$ can be used to show that there is alcohol in the air blown into the multiple reflection system. It can even show what is the concentration of the alcohol. It should be noticed that $\Delta \bar{\sigma_{r}}$ for three independent measurements is not higher than 8%.

## 4. Concept of the Final Device

**Figure 6:**
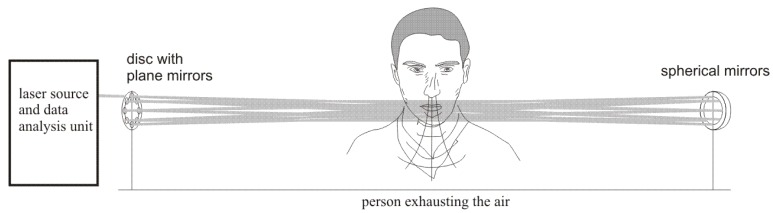
Diagram of the proposed device for alcohol detection in the air exhaled by humans.

Using the analysis and experimental results presented in Section 2 and Section 3, a special device can be developed that could be able to detect the alcohol in the air exhaled by humans. The diagram of proposed device is shown in Figure 6.

**Figure 7:**
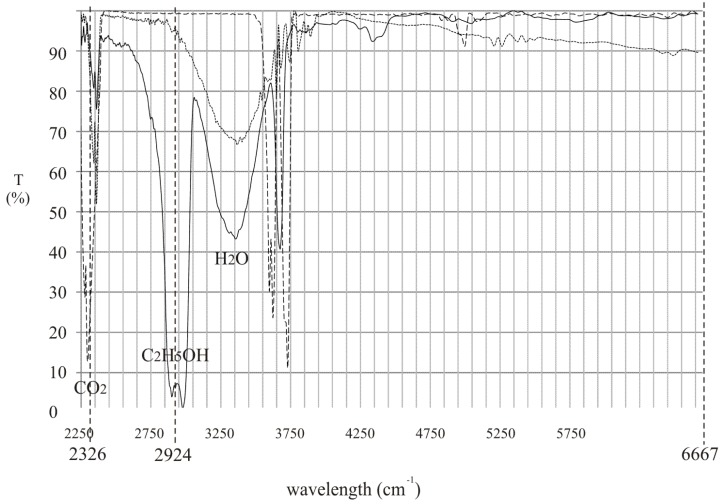
Relation between the transmission spectrum of CO_2_ or H_2_O and alcohol along with the wavelength of laser sources.

To make the proposed device more useful, it should also be able to detect if the person is breathing during the measurements. This problem was already solved and described in patent [14]. Carbon dioxide or water vapor present in the exhaled air, showing that the person is breathing, can be utilized. They can be detected in an analogous way as alcohol. To do this an additional laser source should be used operating at wavelength absorbed by CO_2_ or H_2_O. The relation between the transmission spectrum of the mentioned substances and alcohol along with the wavelength of laser sources is shown in Figure 7.

In the proposed device two inter-band cascade lasers operating at 3.42 µm (2924 cm^−1^) and 4.3 µm (2326 cm^−1^) and laser diode operating at 1.5 µm (6667 cm^−1^) can be used. The working principle can be described using the analysis presented in chapter 2.

Designating the amplitudes of the measured signals at wavelength 3.42 µm, 4.3 µm and 1.5 µm by $S_{1}\left(t\right)$, $S_{2}\left(t\right)$ and $S_{3}\left(t\right)$, respectively, the following equations can be written:

(15) $$s_{ra}\left(t\right)=\frac{S_{3}\left(t\right)-S_{1r}\left(t\right)}{S_{3}\left(t\right)}$$

(16) $$s_{rc}\left(t\right)=\frac{S_{3}\left(t\right)-S_{2r}\left(t\right)}{S_{3}\left(t\right)}$$

where:

$s_{ra}\left(t\right)$ is the relative difference of the amplitude of the reference signal $S_{3}\left(t\right)$ and the amplitude of the normalized signal absorbed by alcohol $S_{1p}\left(t\right)$.
$s_{rc}\left(t\right)$ is the relative difference of the amplitude of the reference signal $S_{3}\left(t\right)$ and the amplitude of the normalized signal absorbed by CO_2_ $S_{2p}\left(t\right)$.

The expressions for $S_{1r}\left(t\right)$ and $S_{2r}\left(t\right)$ are following:

(17) $$S_{1r}\left(t\right)=\frac{\hat{S_{3}}\left(t\right)}{\hat{S_{1}}\left(t\right)}\cdot S_{1}\left(t\right)$$

(18) $$S_{2r}\left(t\right)=\frac{\hat{S_{3}}\left(t\right)}{\hat{S_{2}}\left(t\right)}\cdot S_{2}\left(t\right)$$

where $\hat{S_{1}}\left(t\right)$, $\hat{S_{2}}\left(t\right)$, $\hat{S_{3}}\left(t\right)$ are amplitudes of the signals for lasers operating at 3.42 µm, 4.3 µm and 1.5 µm wavelength, respectively, averaged over a time in which the changes of the laser powers are small in comparison to the changes of alcohol concentration.

The device should calculate $s_{ra}\left(t\right)$ and $s_{rc}\left(t\right)$ over a period in which the changes of the laser power are small in comparison to the changes of alcohol concentration. Based on the calculated $s_{ra}\left(t\right)$ and $s_{rc}\left(t\right)$ their standard deviation $\sigma_{ra}$ and $\sigma_{rc}$ should be estimated. These values of $\sigma_{ra}$ and $\sigma_{rc}$ should be compared with their values calculated for the situation when there is no air exhaled by a person (the relative difference of the amplitudes of the signals are then $s_{ra0}\left(t\right)$ and $s_{rc0}\left(t\right)$ and their standard deviations are $\sigma_{ra0}$ and $\sigma_{rc0}$). Relatively high difference between $\sigma_{rc}$ and $\sigma_{rc0}$ proves that the analysed air is exhaled by humans and the difference between $\sigma_{ra}$ and $\sigma_{ra0}$ shows that there is alcohol in the exhaled air.

It should be noticed that the electromagnetic interference as well as other environmental factors may have some impact on the results of measurements. Therefore, the device should be calibrated for a specific working conditions.

## 5. Conclusions

The analysis and results of the investigations presented in the article shown the possibility to develop a device for stand-off detection of alcohol in the air exhaled by humans. It can be installed at an entrance to a specific area checking every entering person, not causing psychological discomfort. Applying commercially available inter-band cascade lasers the proposed device seems to be relatively simple and not expensive. The device is not able to determine the exact value of alcohol concentration in the exhaled air because of the turbulence in the open space. However, it can designate a person being under the influence of alcohol who, if needed, should be put through an examination using a breathalyzer. It is obvious that the device, if installed at an entrance to a specific area, would significantly increase the throughput of people being checked in a defined period. Thus, the usefulness of the proposed device is unquestionable.

## Figures and Tables

**Table 1:** Averaged amplitudes of the signal for lasers operating at 3.42 µm and 3.55 µm.

Measurement	$\hat{S_{1}}$ (r.u.)	$\hat{S_{2}}$ (r.u.)	$\frac{\hat{S_{2}}}{\hat{S_{1}}}$
1	61,230,420	61,753,928	1.009
2	61,389,675	62,627,976	1.020
3	61,289,652	62,874,508	1.026
4	61,741,613	63,903,865	1.035
5	61,897,609	64,019,494	1.034
6	62,236,705	64,616,967	1.038
7	60,918,372	63,632,695	1.045
8	61,618,170	64,287,179	1.043
9	62,127,678	64,969,336	1.046
10	61,440,914	64,583,786	1.051

**Table 2:** Results of the investigation and calculation based on Equation (11).

*c* (mg/dm^3^)	Measurement	$\hat{S_{1}}$ ×10^−7^ (r.u.)	$\hat{S_{2}}$ ×10^−7^ (r.u.)	*k*	$\hat{\Delta s}$ ×10^4^	$\bar{\Delta s}$ ×10^4^	σ· ×10^4^	$\frac{\sigma }{\bar{\Delta s}}$
0	01	5.59	5.89	1.05367		10.23	27.07	2.65
	1	5.60	5.88		42.20			
	02	5.62	5.94	1.05694				
	2	5.62	5.93		−24.00			
	03	5.61	5.94	1.05882				
	3	5.62	5.95		12.50			
0.30	01	6.17	6.39	1.03566		21.26	10.44	0.49
	1	6.19	6.41		7.58			
	02	6.19	6.40	1.03566				
	2	6.17	6.41		32.90			
	03	6.22	6.46	1.03859				
	3	6.16	6.41		23.30			
0.43	01	6.12	6.18	1.0098		63.40	14.79	0.23
	1	6.18	6.28		71.90			
	02	6.14	6.26	1.01954				
	2	6.09	6.25		75.70			
	03	6.13	6.29	1.0261				
	3	6.17	6.36		42.60			
1.05	01	6.16	6.43	1.04383		41.20	19.40	0.47
	1	6.16	6.46		55.70			
	02	6.21	6.50	1.0467				
	2	6.17	6.50		54.20			
	03	6.14	6.46	1.05212				
	3	6.17	6.50		13.80			
2.32	01	6.08	6.41	1.05428		176.00	70.40	0.40
	1	6.04	6.44		108.00			
	02	6.08	6.40	1.05263				
	2	6.07	6.49		147.00			
	03	5.62	5.84	1.03915				
	3	6.06	6.48		273.00			

**Table 3:** Results of the investigation and calculation based on Equation (14).

*c* (mg/dm^3^)	Measurement	$\hat{S_{1}}$ ×10^−7^ (r.u.)	$\hat{S_{2}}$ ×10^−7^ (r.u.)	$k_{r}$	$\hat{\Delta s_{r}}$ ×10^4^	$\sigma_{r}$ ×10^3^	$\bar{\sigma_{r}}$ ×10^3^	$\Delta \bar{\sigma_{r}}$ ×10^3^	$\frac{\Delta \bar{\sigma_{r}}}{\bar{\sigma_{r}}}$
0	1	5.60	5.88	1.05872	−5.49	1.69	2.05	0.53	0.260
	2	5.62	5.93	1.05441	0.02	1.67			
	3	5.62	5.95	1.06015	0.00	2.80			
0.30	1	6.19	6.41	1.03645	0.04	3.18	3.07	0.21	0.070
	2	6.17	6.41	1.03907	0.03	3.26			
	3	6.16	6.41	1.04102	0.06	2.78			
0.43	1	6.18	6.28	1.01712	0.03	4.09	3.72	0.27	0.070
	2	6.09	6.25	1.02732	0.01	3.62			
	3	6.17	6.36	1.0305	0.09	3.44			
1.05	1	6.16	6.46	1.04969	−0.12	7.47	7.44	0.03	0.005
	2	6.17	6.50	1.05243	−0.20	7.39			
	3	6.17	6.50	1.05360	−0.23	7.45			
2.32	1	6.04	6.44	1.06586	−0.72	15.10	15.37	0.38	0.025
	2	6.07	6.49	1.06838	−0.82	15.10			
	3	6.06	6.48	1.06838	−0.73	15.90			
